# Comorbidity of Narcolepsy and Psychotic Disorders: A Nationwide Population-Based Study in Taiwan

**DOI:** 10.3389/fpsyt.2020.00205

**Published:** 2020-03-25

**Authors:** Jia-Yin Yeh, Yu-Chiau Shyu, Sheng-Yu Lee, Shin-Sheng Yuan, Chun-Ju Yang, Kang-Chung Yang, Tung-Liang Lee, Chi-Chin Sun, Liang-Jen Wang

**Affiliations:** ^1^Department of Child and Adolescent Psychiatry, Kaohsiung Chang Gung Memorial Hospital and Chang Gung University College of Medicine, Kaohsiung, Taiwan; ^2^Community Medicine Research Center, Keelung Chang Gung Memorial Hospital, Keelung, Taiwan; ^3^Institute of Molecular Biology, Academia Sinica, Taipei, Taiwan; ^4^Department of Nursing, Department of Nutrition and Health Sciences, Research Center for Food and Cosmetic Safety, and Research Center for Chinese Herbal Medicine, College of Human Ecology, Chang Gung University of Science and Technology, Taoyuan, Taiwan; ^5^Department of Psychiatry, Kaohsiung Veterans General Hospital, Kaohsiung, Taiwan; ^6^Department of Psychiatry, College of Medicine and Hospital, National Cheng Kung University, Tainan, Taiwan; ^7^Institute of Statistical Science, Academia Sinica, Taipei, Taiwan; ^8^Institute of Biopharmaceutical Sciences, National Yang-Ming University, Taipei, Taiwan; ^9^Department of Radiation Oncology, University of Texas MD Anderson Cancer Center, Houston, TX, United States; ^10^Department of Ophthalmology, Keelung, Chang Gung Memorial Hospital, Keelung, Taiwan

**Keywords:** narcolepsy, comorbidity, epidemiology, psychosis, stimulants

## Abstract

**Background:**

Narcolepsy is a chronic sleep disorder that is likely to have neuropsychiatric comorbidities. Psychotic disorders are characterized by delusion, hallucination, and reality impairments. This study investigates the relationship between narcolepsy and psychotic disorders.

**Design and Methods:**

This study involves patients who were diagnosed with narcolepsy between January 2002 and December 2011 (n = 258) and age- and gender-matched controls (n = 2580) from Taiwan’s National Health Insurance database. Both the patients and the controls were monitored from January 1, 2002 to December 31, 2011 to identify any occurrence of a psychotic disorder. Drugs that have been approved for treating narcolepsy: immediate-release methylphenidate (IR–MPH), osmotic controlled-release formulations of methylphenidate (OROS–MPH), and modafinil, were analyzed. A multivariate logistic regression model was used to evaluate the potential comorbidity of narcolepsy with psychotic disorders.

**Results:**

During the study period, 8.1% of the narcoleptic patients exhibited comorbidity with a psychotic disorder, whereas only 1.5% of the control subjects (1.5%) had psychotic disorders (aOR, 4.07; 95% CI, 2.21–7.47). Of the narcolepsy patients, 41.5, 5.4, and 13.2% were treated with MPH-IR, MPH-OROS, and modafinil, accordingly. Pharmacotherapy for narcolepsy did not significantly affect the risk of exhibiting a psychotic disorder.

**Conclusions:**

This nationwide study revealed that narcolepsy and psychotic disorders commonly co-occur. Pharmacotherapy for narcolepsy was not associated with the risk of psychotic disorders. Our findings serve as a reminder that clinicians must consider the comorbidity of narcolepsy and psychosis.

## Introduction

Narcolepsy is a chronic disabling sleep disorder with an estimated lifetime prevalence of approximately 1 in 2,000 in the general population and it can severely influence daily activities (1, 2). Clinically, it is characterized by excessive daytime sleepiness (EDS) with sudden sleep attacks, rapid eye movement sleep abnormalities such as cataplexy, sleep paralysis, and hypnagogic or hypnopompic hallucinations, nocturnal dyssomnia with fragmented sleep and awakenings (3). The symptoms usually begin in adolescence (4). It has been hypothesized to be caused by a combination of genetic and environmental factors, trigger an autoimmune process that results in hypothalamic destruction, with a loss of hypocretin-1-containing cells, causing the hypocretin deficiency (4, 5). Psychiatric comorbidities with narcolepsy are frequent, and include mood, anxiety, attention deficit hyperactivity, eating disorders, and psychosis (6–9).

Psychotic disorders, consisting of a group of severe mental disorders, are a leading cause of disability globally (10). People with psychotic disorders manifest symptoms of delusion, hallucination, and reality impairment. They suffer from significant functional impairment and have elevated risks of suicide, physical morbidity and premature mortality, loss of productivity, and being difficult to care for caregivers (11, 12). Among narcoleptic symptoms, the hallucinatory episodes, which involve hypnagogic and hypnopompic hallucinations, can be mistaken for an active psychotic state of schizophrenia (13–15). Sleep disturbances, and particularly poor-quality sleep, are also common in schizophrenia (16). Furthermore, the development of narcolepsy in patients with schizophrenia may not be identified because their sleepiness may be attributed to their antipsychotic treatment, and cataplexy may be alleviated or masked by antidopaminergic treatment (17). The overlapping symptoms and similar ages of onset lead to a possibility of misdiagnosis between the two conditions (18–20). These difficulties that are faced by clinicians in correctly diagnosing narcolepsy and psychosis may delay well-targeted treatment.

Modafinil and methylphenidate (MPH) are two effective treatments for narcolepsy. In 1998, the U.S. Food and Drug Administration approved modafinil, an oral agent, for the treatment of EDS that is associated with narcolepsy (21). Modafinil is a wakefulness-promoting drug that probably functions by increasing the extracellular concentration of dopamine and enhancing glutaminergic activity with the result of improved alertness (22). It reacts with histamine and blocks the reuptake of noradrenaline by the noradrenergic terminals on the sleep-promoting neurons from the ventrolateral preoptic nucleus (23). If modafinil cannot be prescribed, then methylphenidate (MPH) is the second-best option. Methylphenidate is a dopamine and catecholamine reuptake inhibitor that also promotes serotonin transmission. MPH reduces EDS, promotes the onset of sleep; increases REM sleep latency, and reduces the percentage of REM sleep (23). However, patients with narcolepsy may develop or experience aggravated psychosis as a consequence of stimulant therapy (such as with methylphenidate or modafinil) (6), although this side-effect seems to be rare even in patients who are treated with high doses of stimulants over a long period (14, 24, 25).

This nationwide population-based analysis was performed to elucidate the co-occurrence rate of narcolepsy and psychotic disorders. The potential effect of pharmacotherapy for narcolepsy on the risk of developing psychotic disorder is also examined.

## Materials and Methods

### Data Source

This retrospective cohort study used data from the ambulatory claims database of the National Health Insurance Research Database of Taiwan (NHIRD-TW), which includes outpatient, ambulatory, hospital inpatient care and dental service data. National Health Insurance (NHI), a compulsory universal health insurance program, was implemented in Taiwan on March 1, 1995. At the end of the year 2000, it covered the delivery of all health care for 22.3 million people in Taiwan (more than 96% of the national population). The Institutional Review Board of Chang Gung Memorial Hospital reviewed and approved this investigation.

In this study, two subjects of the NHIRD-TW, the Longitudinal Health Insurance Database 2000 (LHID2000) and the Longitudinal Health Insurance Database 2005 (LHID2005), were used. They respectively comprise the original claims data for one million beneficiaries that were randomly sampled from the 2000 and 2005 Registries of Beneficiaries of the NHIRD-TW. LHID2000 and LHID2005 include data on all of the medical procedures and prescriptions of two million people (approximately 9% of the population) in Taiwan. A previous study has already demonstrated the reliability of the diagnostic codes in the NHIRD (26–29).

### Study Subjects

The 347.XX codes of the International Classification of Disease, Ninth Revision (ICD-9), were used to identify cases of narcolepsy. A case of narcolepsy was identified by at least one NHI claim record per visit with a diagnosis of narcolepsy (ICD-9 codes: 347). The LHID2000 and LHID2005 database included 258 such cases of narcolepsy (N = 258) between January 1, 2002 and December 31, 2011. The day when narcolepsy was first diagnosed was set as the index date, and the patients’ medical records were traced until a psychotic disorder was diagnosed or until December 31, 2011.

A matching control for each patient in the patient group was selected at random from LHID2000 or LHID2005 using the propensity score matching technique (and a ratio of narcolepsy cases to controls was 1:10). The propensity score was obtained using a multivariable logistic regression model with age and sex as covariates. January 1, 2002 was set as the initial observation date, and the 2,580 control subjects were monitored from the entry date until December 31, 2011 or the diagnosis of a psychotic disorder.

### Variables and Outcomes

The following relevant disorders that were known to be commonly comorbid with narcolepsy were identified in this study (23, 30); attention-deficit hyperactivity disorder (ADHD) (ICD-9-CM code 314.X), autism spectrum disorder (ASD) (ICD-9-CM code 299.X), intellectual disability (ICD-9-CM codes 317 to 319), epilepsy (ICD-9-CM code 345), and alcohol use disorders (ICD-9-CM code 303.X). The results of this study were determined using a diagnosis of any psychotic disorder (ICD-9-CM code 295.X, 297.X, or 298.X).

Medications were identified using the Anatomical Therapeutic Chemical (ATC) classification system. According to the Food and Drug Administration of Taiwan, only three drugs were licensed for treating narcolepsy before 2011; they were immediate-release methylphenidate (IR–MPH) (ATC code N06BA04), osmotic controlled-release formulations of methylphenidate (OROS–MPH) (ATC code N06BA04), and modafinil (ATC code N06BA07). These drugs are restricted by the Bureau of NHI in Taiwan, and ideally any prescription for narcolepsy is recorded in an ambulatory care, pharmacy, or hospital care claim.

### Statistical Analysis

To study the distribution of the study population, the characteristics of the narcolepsy group were compared with those of the control group using a chi-square (χ^2^) test or *t*-test. To compare the ages at the diagnosis of a psychotic disorder between the two groups, a Mann-Whitney U test was conducted. A multivariate logistic regression model was used to evaluate the potential comorbidity of a psychotic disorder with narcolepsy across all study subjects. Multivariate logistic regression was also used to investigate the potential effect of a pharmacotherapy prescription for narcolepsy on the risk of a psychotic disorder among narcoleptic patients. The results are presented as an adjusted odds ratio (aOR) and 95% confidence intervals (CIs). All data management and statistical analyses were carried out using the Statistical Package for the Social Sciences (SPSS) Version 16.0 (SPSS Inc., Chicago, IL, USA). A two-tailed value of *p <* 0.05 indicated statistical significance.

## Results

**Table 1** presents the characteristics of the narcolepsy group and the control group. No significant difference in age and sex between the two groups. The narcolepsy group was more likely than the control group to exhibit comorbidities among ADHD (8.9%), ASD (2.3%), intellectual disability (2.3%), and epilepsy (8.9%). Of patients with narcolepsy, 41.5, 5.4, and 13.2% were prescribed MPH-IR, MPH-OROS, and modafinil, respectively. In the monitoring period, 8.1% of the cases of narcolepsy were comorbid with a psychotic disorder. In contrast, 1.5% of the control subjects had a psychotic disorder. The age at diagnosis of a psychotic disorder was lower for narcoleptic patients (29.4 years) than for the controls (36.3 years).

**Table 1:** Characteristics of patients with narcolepsy and control subjects in Taiwan from 2002 to 2011.

Characteristics	Narcolepsy (N = 258)	Controls (N = 2,580)	Statistics	*P*-value
**Age (years)**	29.3 ± 20.4	30.2 ± 20.9	0.60 ^a^	0.552
**Gender**			0.47 ^b^	0.495
Female	118 (45.7)	1,123 (43.5)		
Male	140 (54.3)	1,457 (56.5)		
**Comorbidity**				
ADHD	23 (8.9)	24 (0.9)	91.81 ^b^	< 0.001*
ASD	6 (2.3)	6 (0.2)	24.40 ^b^	< 0.001*
Intellectual disability	6 (2.3)	19 (0.7)	6.78 ^b^	0.009*
Epilepsy	23 (8.9)	38 (1.5)	61.76 ^b^	< 0.001*
**Medication treatment**				
Methylphenidate-IR	107 (41.5)	23 (0.9)	883.72 ^b^	< 0.001*
ethylphenidate-OROS	14 (5.4)	7 (0.3)	84.86 ^b^	< 0.001*
Modafinil	34 (13.2)	0 (0)	344.12 ^b^	< 0.001*
**Diagnosed a psychotic disorder**	21 (8.1)	39(1.5)	49.79 ^b^	< 0.001*
Age at diagnosis (years)	29.4 ± 21.2	36.3 ± 17.5	2.08 ^c^	0.038*

Data is expressed by n (%) or mean ± SD; Statistic values: ^a^ t using an independent t-test; ^b^ Pearson’s χ^2^; ^c^ Z using Mann-Whitney U test; ADHD, attention-deficit hyperactivity disorder; ASD, Autism Spectrum Disorder; Age, age at diagnosis or at recruitment; *P < 0.05.

The multivariate logistic regression models (**Table 2**) indicate that patients with narcolepsy were more likely than the control subjects to be diagnosed with a psychotic disorder (aOR, 4.07; 95% CI, 2.21–7.47). Moreover, subjects who were diagnosed with ASD (aOR, 8.13; 95% CI, 1.74–38.05), intellectual disability (aOR, 4.33; 95% CI, 1.05–17.90), and epilepsy (aOR, 5.15; 95% CI, 2.23–11.93) had a higher risk of being diagnosed with a psychotic disorder.

**Table 2:** Logistic regression models for the risk of diagnosis with a psychotic disorder among all participants, controlling for gender, age, and comorbidities.

Variables	Psychotic disorder		
	n (%)	aOR (95% CI)	*P*-value
**Diagnostic group**			
Controls (N = 2,580)	39 (1.5)	1	
Narcolepsy (N = 258)	21 (8.1)	4.07 (2.21–7.47)	<0.001*
**Age at recruitment**	–	1.01 (1.00–1.02)	0.156
**Gender**			
Male (N = 1,241)	33 (2.1)	1	
Female (N = 1,597)	27 (2.2)	0.99 (0.58–1.70)	0.965
**ADHD**			
Without (N = 2,791)	53 (1.9)	1	
With (N = 47)	7 (14.9)	1.74 (0.51–5.87)	0.373
**ASD**			
Without (N = 2,826)	55 (1.9)	1	
With (N = 12)	5 (41.7)	8.13 (1.74–38.05)	0.008*
**Intellectual disability**			
Without (N = 2,813)	55 (2.0)	1	
With (N = 25)	5 (20.0)	4.33 (1.05–17.90)	0.043*
**Epilepsy**			
Without (N = 2,777)	50 (1.8)	1	
With (N = 61)	10 (16.4)	5.15 (2.23–11.93)	<0.001*

ADHD, attention-deficit hyperactivity disorder; ASD, Autism Spectrum Disorder; aOR, adjusted odds ratios; 95% CI, 95% confidence interval; n, number of diagnosed psychotic disorders. *P < 0.05.

**Table 3** provides the effects of pharmacotherapy for narcolepsy on a comorbid psychotic disorder. Prescription of methylphenidate or modafinil for the narcoleptic patients was not significantly associated with a psychotic disorder. However, patients with ASD had a higher risk of having a psychotic disorder (aOR, 10.42; 95% CI, 1.40–77.52).

**Table 3:** Risk factors of diagnosis with a psychotic disorder in narcoleptic patients.

Variables	Psychotic disorder		
	n (%)	aOR (95% CI)	*P*-value
**Age at recruitment**	–	1.00 (0.98–1.03)	0.755
**Gender**			
Male (N = 140)	12 (8.6)	1	
Female (N = 118)	9 (7.6)	1.00 (0.38–2.66)	1.000
**ADHD**			
Without (N = 235)	16 (6.8)	1	
With (N = 23)	5 (21.7)	1.21 (0.26–5.59)	0.812
**ASD**			
Without (N = 252)	18 (7.1)	1	
With (N = 6)	3 (50.0)	10.42 (1.40–77.52)	0.022*
**Intellectual disability**			
Without (N = 252)	19 (7.5)	1	
With (N = 6)	2 (33.2)	3.25 (0.38–27.68)	0.280
**Epilepsy**			
Without (N = 235)	17 (7.2)	1	
With (N = 23)	4 (17.4)	2.02 (0.53–7.67)	0.302
**Methylphenidate**			
Without (N = 151)	9 (6.0)	1	
With (N = 107)	12 (11.2)	2.39 (0.83–6.86)	0.106
**Modafinil**			
Without (N = 224)	19 (8.5)	1	
With (N = 34)	2 (5.9)	0.59 (0.11–3.04)	0.528

ADHD, attention-deficit hyperactivity disorder; ASD, Autism Spectrum Disorder; aOR, adjusted odds ratios; 95% CI, 95% confidence interval; n, number of diagnosed psychotic disorders. *P < 0.05.

We further analyzed the individual effect of IR-MPH, OROS-MPH, and modafinil on psychotic disorders (**Supplementary Table 1**). We also examined that association between the risk of psychotic disorders, antipsychotic drugs, antidepressant drugs, and alcohol use disorders. We found that prescription of IR-MPH, OROS-MPH, or modafinil for the narcoleptic patients was not significantly associated with a psychotic disorder. However, prescription of antipsychotic drugs was associated with a higher risk of having a psychotic disorder (aOR, 12.65; 95% CI, 2.90–55.13).

## Discussion

### The Prevalence of Narcolepsy

According to our study, the prevalence of narcolepsy in Taiwan is about 12.9 per 100,000, slightly lower than the global mean prevalence of approximately 30 per 100,000 (31). To our knowledge, reliable evidence about the prevalence of narcolepsy in Taiwan is still lacking currently. In Asia, the prevalence in Korea and China is 15 and 30 per 100,000, respectively (31). For incidence rates, Dodd et al. (32) reported that narcolepsy incidence in Taiwan was 0.29 per 100,000 person-years, relatively lower than those in European Union and North America. The lower incidence/prevalence of narcolepsy in Taiwan may be related to under diagnosis or under treatment. Moreover, polymorphism of HLA-DQB1*0602, H1N1 influenza vaccination, and H1N1 infection itself have been associated with narcolepsy susceptibility (31) (33). Various genetic or environment factors may also contribute to the discrepancies in prevalence of narcolepsy across countries.

### Narcolepsy and the Increased Risk of a Psychotic Disorder

This nationwide, population-based cohort study revealed that narcolepsy was associated with a four-fold greater risk of a psychotic disorder. Most relevant studies have discussed the risk of psychosis rather than the psychotic illness among narcoleptic patients.

An association between psychosis and narcolepsy has been reported in both adults and children with frequencies in the range 1 to 10% (18, 19, 25, 34, 35). A cross-sectional observational study of 28 narcolepsy and 21 schizophrenia patients found that narcoleptic and schizophrenic patients did not differ with respect to frequency or sensory modality of hallucinations. It also found that the lifetime prevalence of hallucinations did not differ between individuals with schizophrenia and those with narcolepsy (36). Some reports have found narcolepsy patients with challenging differential diagnoses of psychotic disorders (37, 38). Diagnostic and therapeutic challenges exist for patients with comorbid narcolepsy and psychotic disorder owing to their similar onset times (usually in early life and often in childhood) and their overlapping symptoms (13, 14, 19, 37–41). However, our cohort revealed that the age at diagnosis of a psychotic disorder was lower for narcoleptic patients (29.4 years) than for the controls (36.3 years). There were some possible explanations for this. First, patients have dual diagnoses of narcolepsy and schizophrenia have strong biological tendency and tend to onset earlier than patients who had schizophrenia only. The second explanation is that the psychotic disorders among patients who had narcolepsy were diagnosed earlier due to their hypnogogic or hypnopompic hallucination.

A cohort study found that narcolepsy is not an under-recognized disease in adult patients with schizophrenia or schizoaffective disorder (42). It also indicated a possible age-dependent relationship between schizophrenia and narcolepsy, with patients who initially experienced narcolepsy in childhood or early adolescence at a higher risk of developing schizophrenia (6, 42). However, the study did not consider the effects of psychotic disorders on risk of narcolepsy. Additionally, its exclusion of patients who were considered to be unable to answer a questionnaire might have generated bias, such as by the failure to include patients with possibly highly severe functional impairment.

Numerous pathophysiological mechanisms potentially explain the high comorbidity of narcolepsy and psychotic disorder. Dopaminergic neurons in ventral tegmental area (VTA) contribute to the regulation of sleep-awake cycle and are involved in the mechanism of cataplexy (43). Hypocretinergic neurotransmission regulates dopamine firing in ascending midbrain neurons in the ventral tegmental area and prefrontal cortex, and possibly at the level of the nucleus accumbens, which are critical areas in schizophrenia pathophysiology. Therefore, hypocretin, which is known to be an important cause of narcolepsy (4), may also influence dopamine signaling, causing dysfunction of the mesocorticolimbic and mesocorticostriatal circuits, which are the two main pathways that are implicated in the pathophysiology of psychotic symptoms (44).

Some reports have suggested an overlapping autoimmune pathogenesis between narcolepsy and schizophrenia-like psychosis, associated with both HLA and autoantibodies (34, 35, 45–47). An autoimmune basis of schizophrenia has been suggested (48), and several studies have examined investigated its association with the HLA region and DQ6 alleles, particularly of the DQB1*0602 subtype (45–47). Narcolepsy is also strongly associated with the HLA DQB1 * 0602 subtype, which is found in up to 98% of patients (36).

Previous studies have studied a potential autoimmune basis of the relationship between schizophrenia and narcolepsy by assessing NMDAR autoantibodies in either the serum or CSF of patients with anti-NMDA encephalitis, but discordant results have been obtained with respect to narcolepsy (6, 34, 35). Some environmental clues about autoimmunity also suggest the overlapping pathogeneses of narcolepsy and psychosis. Several upper airway infections (streptococcal and H1N1 influenza) and Pandemrix vaccinations have been reported as triggering narcolepsy (49). Prenatal maternal infection with herpes simplex virus type 2, influenza, or cytomegalovirus has been associated with the risk of adult schizophrenia (50). Early-life infections that are caused by viruses or bacteria have also been associated with the risk of adult psychotic illness (51, 52).

A recent review study indicates that severe sleep deprivation itself may cause psychosis (53). Within 24–48 h of sleep deprivation, perceptual distortions and depersonalization occur. After 48–90 h without sleep, complex hallucinations and delusion develop, yielding psychotic states indistinguishable from acute psychosis or toxic delirium. The underlying biological mechanism may be neuronal instability or a related defect in neural transmission, especially cholinergic, and central chromatolysis (53).

### Pharmacotherapy and Psychotic Disorder in Narcoleptic Patients

Our data do not support an association between the use of MPH or modafinil and the development of incident psychotic disorder. Among adolescents and young adults with ADHD who were receiving prescription stimulants, new-onset psychosis occurred in approximately 1 in 660 patients. Amphetamine use was associated with a greater risk of psychosis than MPH (54). A study revealed that initiation of MPH treatment did not increase the risk of psychotic events in adolescents and young adults, including in those individuals with a history of psychosis (55). Few investigations have found that a high dose of modafinil may cause mania and psychosis in patients with medical or psychiatric diseases and those who are shift-workers (56, 57). However, in a recent study, modafinil-induced psychosis was identified in only a few case reports (58, 59).

In the present study, MPH users among patients with narcolepsy were more likely than others to have a psychotic disorder but not significantly so (OR = 2.39, *P*-value = 0.106). We further analyzed the individual effect of IR-MPH, OROS-MPH, and modafinil on development of psychosis, and there was still no significant association between drug treatment and psychotic disorders. A self-controlled case series revealed no increased risk of incident psychotic events during MPH-exposure relative to periods of non-exposure (incidence rate ratio: 1.02, 95%: 0.53–1.97) (60). Nonetheless, the prevalence of MPH-induced psychosis has been reported as 18.2% among MPH-treated patients, and this effect should not be considered rare (6, 24, 25). Notably, previous studies demonstrated a significantly higher occurrence of psychosis, substance misuse, and psychiatric hospitalizations in patients using high-dose stimulants compared to those using standard doses (24, 25). However, the doses of medication were not available in this study. Further investigations with a larger sample size and comprehensive information about stimulants prescription are warranted to clarify the relationship between stimulants doses and psychotic disorders.

One study reviewed data from 49 randomized controlled clinical trials, analyzed the association between psychotic symptoms and the use of ADHD drugs, and found a rate of psychosis/mania events of 1.48 per 100 person-years in the ADHD treatment group. However, only two events were identified in trials of modafinil and four in trials of MPH (61). We found that prescription of antipsychotic drugs, but not antidepressant drugs or alcohol use disorders, was associated with a higher risk of having a psychotic disorder. This is not surprising because patients who suffered from psychotic symptoms may be prescribed antipsychotic drugs for treating psychotic symptoms.

A recent nation-wide study comprised 789 patients with schizophrenia suggested that the use of CNS stimulant (including modafinil and methylphenidate) may reduce in the overall number of psychiatric bed-days (62). Although CNS stimulant use is not recommended in any treatment guidelines for psychosis, it may be beneficial for cognition, negative symptoms, and the overall function of the patient with schizophrenia. Positive symptoms of schizophrenia are known to be caused by excess dopamine in the midbrain and substantia nigra. CNS stimulants, such as modafinil, may only increase dopamine in the prefrontal cortex. The brain target site influenced by CNS stimulant is different from that for pathophysiology of schizophrenia may count for the possible mechanism that CNS stimulants not seem to enhance psychosis (63, 64).

### Comorbidities and an Increased Risk of Psychotic Disorder

In this investigation, intellectual disability and epilepsy were risk factors for psychotic disorder. Various studies have demonstrated that intellectual disability and epilepsy are risk factors for comorbidity of a psychotic disorder. A cohort study of people with intellectual disability and the general population found an increased diagnosis of psychotic disorders among patients with intellectual disability (OR:10.4) (65).

A systemic review found that individuals with epilepsy have approximately an eight-fold greater risk of psychosis than those without and 6% of them have a comorbid psychotic illness with an even higher prevalence of psychosis in cases of temporal lobe epilepsy (7%) (66). The neurotoxic effect of epilepsy, a “kindling” process by which acute seizure discharges, a “forced normalization” process, on-going subictal activity in the limbic system, antiepileptic medication, similar structural brain abnormalities and genetic abnormalities in patients with schizophrenia and patients with epilepsy, all may explain the high comorbidity (66).

According to recent studies, ASD seems not to be a common comorbidity of narcolepsy. Our research identified only five cases of concomitant narcolepsy and ASD (67, 68). However, around 2.33% of the patients with narcolepsy had also been diagnosed with ASD in our study. Patients with narcolepsy and ASD were found to have a higher risk of psychotic disorder than those with only narcolepsy. Owing to the few data on the association between narcolepsy and ASD, the pathophysiological pathway of the co-occurrence remains unclear. In narcoleptic patients, ASD may be an additional neurodevelopmental disorder (67).

Although ASD is now believed to be distinct from psychotic disorders, they share multiple phenotypic similarities and risk factors, and reportedly co-occur. Psychotic disorder rates of patients with ASD range from 5 to 16% (69). Several models have been used explain the association and comorbidity between psychotic disorders and ASD. They include a potential autoimmune basis for a relationship among narcolepsy, ASD and psychotic disorder.

Maternal mild acute inflammation that is caused by infection, including influenza, triggers abnormal activation of microglia and overexpression of inflammatory cytokines. This reaction has been observed in animal models. This maternal immune activation generates imbalanced cytokine profiles and characteristics of the offspring that are similar to ASD and schizophrenia (70). The overlapping pathophysiological mechanisms (49) potentially explain the association among narcolepsy, ASD, and psychotic disorder.

### Strengths and Limitations

To the best of our knowledge, this study is the first to investigate the association between narcolepsy and psychotic disorders, and the first to elucidate the relationship between psychotic disorders and pharmacotherapy in narcoleptic patients. The strengths of the investigation include its use of a nationwide population-based database to provide a nationally representative sample, an appropriate observation period, physician-based diagnoses to identify cases of narcolepsy and psychotic disorder, and clear information about the prescription of medications. The use of registration of medical claim data from the NHIRD prevents recall bias and selection bias.

However, this investigation has certain limitations. First, misclassifications may arise from the fact that the diagnoses of narcolepsy and psychotic disorders were taken from the registration database rather than mad using validated structural diagnostic instruments. Patients with idiopathic hypersomnia or other hypersomnolence disorders may have been coded as narcoleptics. Besides, the ICD-9-CM does not differentiate between type 1 and type 2 narcolepsy. Moreover, the diagnostic codes for narcolepsy and psychotic disorders in the NHIRD have not been validated, although the accuracy of disease diagnoses in the National Health Insurance system in 1990 to 1991 was reportedly between 54 and 85% (71), and the validity of diagnosis codes in the NHIRD has been confirmed in previous studies (26–29). Second, previous studies have examined many comorbidities of narcolepsy, but this study considered only ADHD, ASD, intellectual disability, and epilepsy. Other confounding factors, such as other organic diseases and psychiatric disorders, may have had an unrecognized effect. Third, the NHIRD is an administrative database that does not contain comprehensive clinical information, such as medication compliance and laboratory examination data. Therefore, our results merely reveal increased associations between psychotic disorders and narcolepsy and no increased associations between psychotic disorders and MPH or modafinil use in narcoleptic patients. This investigation did not analyze whether a psychiatric condition persisted following the diagnosis of narcolepsy or discontinuation of relevant medication. Hence, it could not establish a causal relationship and further longitudinal research is warranted. Finally, this study was carried out among Taiwan’s population, and therefore its generalizability to other countries requires further investigation. Besides, the prevalence of narcolepsy varies in different countries and ethnic groups (31). However, we were unable to control for ethnicity because such information was not registered in the NHIRD. With increasing immigrants in Taiwan, such bias influenced by the different susceptibility to narcolepsy across ethnicity might require other form of study.

## Conclusion

This population-based longitudinal study provides evidence that patients with narcolepsy had higher rates of psychotic disorders than a control group. Among narcoleptic patients, the prescription of MPH or modafinil was not associated with a psychotic disorder. However, narcoleptic patients with ASD were found to have a higher risk of a psychotic disorder than those without. Further comprehensive research is warranted to enhance the public awareness of narcolepsy and the possible co-occurrence of psychotic disorders, especially those comorbid with ASD. Continuous efforts must also be made to identify exactly the associated pathophysiological mechanisms and the causal relationship between narcolepsy and psychotic disorders.

## Data Availability Statement

The datasets for this manuscript are not publicly available because: The data are provided by the National Health Insurance Administration, Ministry of Health and Welfare and managed by the National Health Research Institutes. Requests to access the datasets should be directed to nhird@nhri.org.tw.

## Ethics Statement

This study has been approved by the institutional review board at Chang Gung Memorial Hospital.

## Author Contributions

J-YY and Y-CS are joint first authors and contribute equally to this manuscript. J-YY participated in reviewing references and drafting the manuscript. Y-CS executed the statistical analysis. S-YL, S-SY, C-JY, K-CY, T-LL, and C-CS participated in the design of the study. L-JW interpreted the data and revised the manuscript. All authors read and approved the final manuscript and contributed to the drafting and revising of the paper.

## Funding

This study was sponsored by the Chang Gung Memorial Hospital Research Projects (CMRPG8D0581, CMRPG2G0071, CLRPG2C0023, and CGRPG2F0021).

## Conflict of Interest

The authors declare that the research was conducted in the absence of any commercial or financial relationships that could be construed as a potential conflict of interest.
