# Supplementary material for: Comorbidity of Narcolepsy and Psychotic Disorders: A Nationwide Population-Based Study in Taiwan
Source: Front Psychiatry. 2020 Mar 25;11:205. doi: 10.3389/fpsyt.2020.00205 (PMC7109289; doi:10.3389/fpsyt.2020.00205)
Supplement: Supplementary file 1 [file Table_1.doc]

**Supplementary Table 1** Risk factors of diagnosis with a psychotic disorder in narcoleptic patients

|  |  | **Model 1** |  |
| --- | --- | --- | --- |
|  | n (%) | aOR (95% CI) | *P*-value |
| **Age at recruitment** | - | 1.00 (0.96-1.03) | 0.728 |
| **Gender** |  |  |  |
| Male (N=140) | 12 (8.6) | 1 |  |
| Female (N=118) | 9 (7.6) | 1.00 (0.35-2.82) | 0.991 |
| **ADHD** |  |  |  |
| Without (N=235) | 16 (6.8) | 1 |  |
| With (N=23) | 5 (21.7) | 2.27 (0.35-14.68) | 0.391 |
| **ASD** |  |  |  |
| Without (N=252) | 18 (7.1) | 1 |  |
| With (N=6) | 3 (50.0) | 14.65 (1.41-152.18) | 0.025* |
| **Intellectual disability** |  |  |  |
| Without (N=252) | 19 (7.5) | 1 |  |
| With (N=6) | 2 (33.2) | 4.82 (0.47-49.81) | 0.187 |
| **Epilepsy** |  |  |  |
| Without (N=235) | 17 (7.2) | 1 |  |
| With (N=23) | 4 (17.4) | 1.52 (0.32-7.32) | 0.601 |
| **Alcohol use disorders** |  |  |  |
| Without (N=254) | 20 (7.9) | 1 |  |
| With (N=4) | 1 (25.0) | 5.71 (0.35-92.42) | 0.220 |
| **MPH-IR** |  |  |  |
| Without (N=151) | 9 (6.0) | 1 |  |
| With (N=107) | 12 (11.2) | 1.81 (0.54-6.06) | 0.336 |
| **MPH-OROS** |  |  |  |
| Without (N=244) | 19 (7.8) | 1 |  |
| With (N=14) | 2 (14.3) | 0.38 (0.03-4.47) | 0.442 |
| **Modafinil** |  |  |  |
| Without (N=224) | 19 (8.5) | 1 |  |
| With (N=34) | 2 (5.9) | 1.06 (0.18-6.21) | 0.946 |
| **Antipsychotic drugs** |  |  |  |
| Without (N=163) | 4 (2.5) | 1 |  |
| With (N=95) | 17 (17.9) | 12.65 (2.90-55.13) | 0.001* |
| **Antidepressant drugs** |  |  |  |
| Without (N=186) | 10 (5.4) | 1 |  |
| With (N=72) | 11 (15.3) | 1.44 (0.45-4.58) | 0.536 |

Abbreviations: ADHD, attention-deficit hyperactivity disorder; ASD, Autism Spectrum Disorder; aOR, adjusted odds ratios; 95% CI, 95% confidence interval; n, number of diagnosed psychotic disorders. **P*<0.05
